# Actual and Missed Opportunities for End-of-Life Care Discussions With Oncology Patients: A Qualitative Study

**DOI:** 10.1001/jamanetworkopen.2021.13193

**Published:** 2021-06-10

**Authors:** Kristin E. Knutzen, Olivia A. Sacks, Olivia C. Brody-Bizar, Genevra F. Murray, Raina H. Jain, Lindsay A. Holdcroft, Shama S. Alam, Matthew A. Liu, Kathryn I. Pollak, James A. Tulsky, Amber E. Barnato

**Affiliations:** 1 Department of Behavioral, Social, and Health Education Sciences, Rollins School of Public Health, Emory University, Atlanta, Georgia; 2 Department of Surgery, Boston Medical Center, Boston, Massachusetts; 3 Dartmouth College, Hanover, New Hampshire; 4 Department of General Medicine, Boston Medical Center, Boston, Massachusetts; 5 Geisel School of Medicine, Dartmouth College, Hanover, New Hampshire; 6 Pharmaceutical Product Development, Evidera, Bethesda, Maryland; 7 School of Medicine, University of California, San Diego, La Jolla; 8 School of Medicine, Duke University, Durham, North Carolina; 9 Department of Psychosocial Oncology and Palliative Care, Dana-Farber Cancer Institute, Boston, Massachusetts; 10 Division of Palliative Medicine, Department of Medicine, Brigham and Women’s Hospital, Boston, Massachusetts; 11 The Dartmouth Institute for Health Policy and Clinical Practice, Geisel School of Medicine at Dartmouth, Lebanon, New Hampshire; 12 Norris Cotton Cancer Center, Dartmouth-Hitchcock Medical Center, Lebanon, New Hampshire

## Abstract

**Importance:**

Early discussion of end-of-life (EOL) care preferences improves clinical outcomes and goal-concordant care. However, most EOL discussions occur approximately 1 month before death, despite most patients desiring information earlier.

**Objective:**

To describe successful navigation and missed opportunities for EOL discussions (eg, advance care planning, palliative care, discontinuation of disease-directed treatment, hospice care, and after-death wishes) between oncologists and outpatients with advanced cancer.

**Design, Setting, and Participants:**

This study is a secondary qualitative analysis of outpatient visits audio-recorded between November 2010 and September 2014 for the Studying Communication in Oncologist-Patient Encounters randomized clinical trial. The study was conducted at 2 US academic medical centers. Participants included medical, gynecological, and radiation oncologists and patients with stage IV malignant neoplasm, whom oncologists characterized as being ones whom they “…would not be surprised if they were admitted to an intensive care unit or died within one year.” Data were analyzed between January 2018 and August 2020.

**Exposures:**

The parent study randomized participants to oncologist- and patient-directed interventions to facilitate discussion of emotions. Encounters were sampled across preintervention and postintervention periods and all 4 treatment conditions.

**Main Outcomes and Measures:**

Secondary qualitative analysis was done of patient-oncologist dyads with 3 consecutive visits for EOL discussions, and a random sample of 7 to 8 dyads from 4 trial groups was analyzed for missed opportunities.

**Results:**

The full sample included 141 patients (54 women [38.3%]) and 39 oncologists (8 women [19.5%]) (mean [SD] age for both patients and oncologists, 56.3 [10.0] years). Of 423 encounters, only 21 (5%) included EOL discussions. Oncologists reevaluated treatment options in response to patients’ concerns, honored patients as experts on their goals, or used anticipatory guidance to frame treatment reevaluation. In the random sample of 31 dyads and 93 encounters, 35 (38%) included at least 1 missed opportunity. Oncologists responded inadequately to patient concerns over disease progression or dying, used optimistic future talk to address patient concerns, or expressed concern over treatment discontinuation. Only 4 of 23 oncologists (17.4%) had both an EOL discussion and a missed opportunity.

**Conclusions and Relevance:**

Opportunities for EOL discussions were rarely realized, whereas missed opportunities were more common, a trend that mirrored oncologists’ treatment style. There remains a need to address oncologists’ sensitivity to EOL discussions, to avoid unnecessary EOL treatment.

## Key Points

**Question:**

How do oncologists successfully navigate and miss opportunities for discussions about end of life (EOL), including advance care planning, palliative care, discontinuation of disease-directed treatment, hospice care, and after-death wishes, with outpatients with advanced cancer?

**Findings:**

In this secondary qualitative analysis of 423 outpatient encounters, only 21 encounters (5%) included EOL discussions, whereas a random sample of 93 encounters revealed that 35 encounters (38%) included missed opportunities for EOL discussions. Oncologists missed opportunities for these discussions when they responded inadequately to patient concerns about disease progression or dying, used optimistic future talk to address patient concerns, or expressed concern over treatment discontinuation.

**Meaning:**

Opportunities for EOL discussions were rarely realized, and missed opportunities for these discussions were common, a trend that seemed to mirror oncologists’ treatment style.

## Teaser

This qualitative study describes successful navigation and missed opportunities for end-of-life discussions, including advance care planning, palliative care, discontinuation of disease-directed treatment, hospice care, and after-death wishes, between oncologists and outpatients with advanced cancer.

## Introduction

End-of-life (EOL) treatment that is discordant with patient goals can negatively affect patients, loved ones, and health care systems and represents a major public health problem.^1^ The American Society of Clinical Oncology and National Quality Forum (NQF) have identified discordant treatment for patients with cancer as receipt of chemotherapy during the last 14 days of life (NQF measure 0210), intensive care unit admission during the last 30 days of life (NQF measure 0213), nonreferral to hospice (NQF measure 0215), or late referral to hospice (NQF measure 0216).^2^ Such treatment adversely affects patient quality of life, quality of dying, and caregiver bereavement outcomes.^3,4,5,6^ This treatment can often be avoided when patients and clinicians discuss goals of care.

Clinical practice guidelines^7,8^ recommend that oncology practitioners initiate early discussion of goals of care with patients with a life expectancy of less than 1 year. Early initiation of these conversations elicits goals from seriously ill patients, guiding decision-making and avoiding aggressive, burdensome, and unnecessary EOL treatment. Because oncologists often see patients across disease trajectories—building long-term relationships and rapport—they have unique opportunities to initiate early and continued conversations around goals of care.^9^ However, most discussions of EOL preferences do not occur until approximately 1 month before death, despite most patients desiring this information earlier.^10^ Understanding the frequency and nature of EOL discussions between oncologists and outpatients with advanced cancer can inform future interventions to promote more effective communication.^11^

We identified 5 key EOL components. Advance care planning (ACP), palliative care, discontinuation of disease-directed treatment, and hospice care were identified deductively, because we believe that these are directly associated with the aforementioned NQF measures. The component of after-death wishes was identified inductively from encounter review. In this qualitative analysis, we sought to characterize how these EOL components were both successfully navigated and neglected (missed opportunities) in unstructured outpatient-oncologist encounters.

## Methods

This study was approved by the Dartmouth College Committee for the Protection of Human Subjects. Written, informed consent was obtained from all participants. The methods used by this study adhered to the Consolidated Criteria for Reporting Qualitative Research (COREQ) reporting guideline for reporting qualitative research.^12^

This is a secondary qualitative analysis of outpatient oncology visits at 2 US academic medical centers, audio-recorded between November 2010 and September 2014 for the Studying Communication in Oncologist-Patient Encounters randomized clinical trial. Details of the trial have been previously described.^13,14^ The parent trial aimed to improve communication between patients with advanced cancer and their oncologists. Patients were randomized to 4 study groups, stratified by site and sex. Eligible practitioners included medical, gynecological, and radiation oncologists. Eligible patients included those with stage IV malignant neoplasm of whom their oncologist said they “…would not be surprised if they were admitted to an intensive care unit or died within one year.” Three consecutive encounters between each patient-oncologist dyad were audio-recorded, including 1 baseline conversation before treatment intervention and 2 conversations recorded after the intervention. *Dyad* describes a patient-oncologist pair, although other individuals, such as family members or other health care practitioners, were often present during encounters.

Secondary qualitative data analysis was performed between January 2018 and August 2020. We outline sampling measures in the Figure. We restricted analysis to dyads for which there were no missing data (to preserve the longitudinal aspect of these conversations) and the participating oncologist was present at each encounter (eg, we excluded dyads if ≥1 of the 3 encounters involved only an advance practice practitioner). The full sample refers to 423 encounters among 141 patient-oncologist dyads. The random sample refers to a sample of 7 or 8 patient-oncologist dyads from each of the 4 trial groups (31 dyads and 93 encounters). All encounters in the random sample were professionally transcribed. In the remaining encounters, only those with EOL discussions were transcribed. For our purposes, EOL discussions included any mention (ie, passing mention without further discussion) and substantial discussion (ie, full discussion of any EOL topics and potential influence on patient disease trajectory, quality of life, or treatment preferences).

**Figure.**
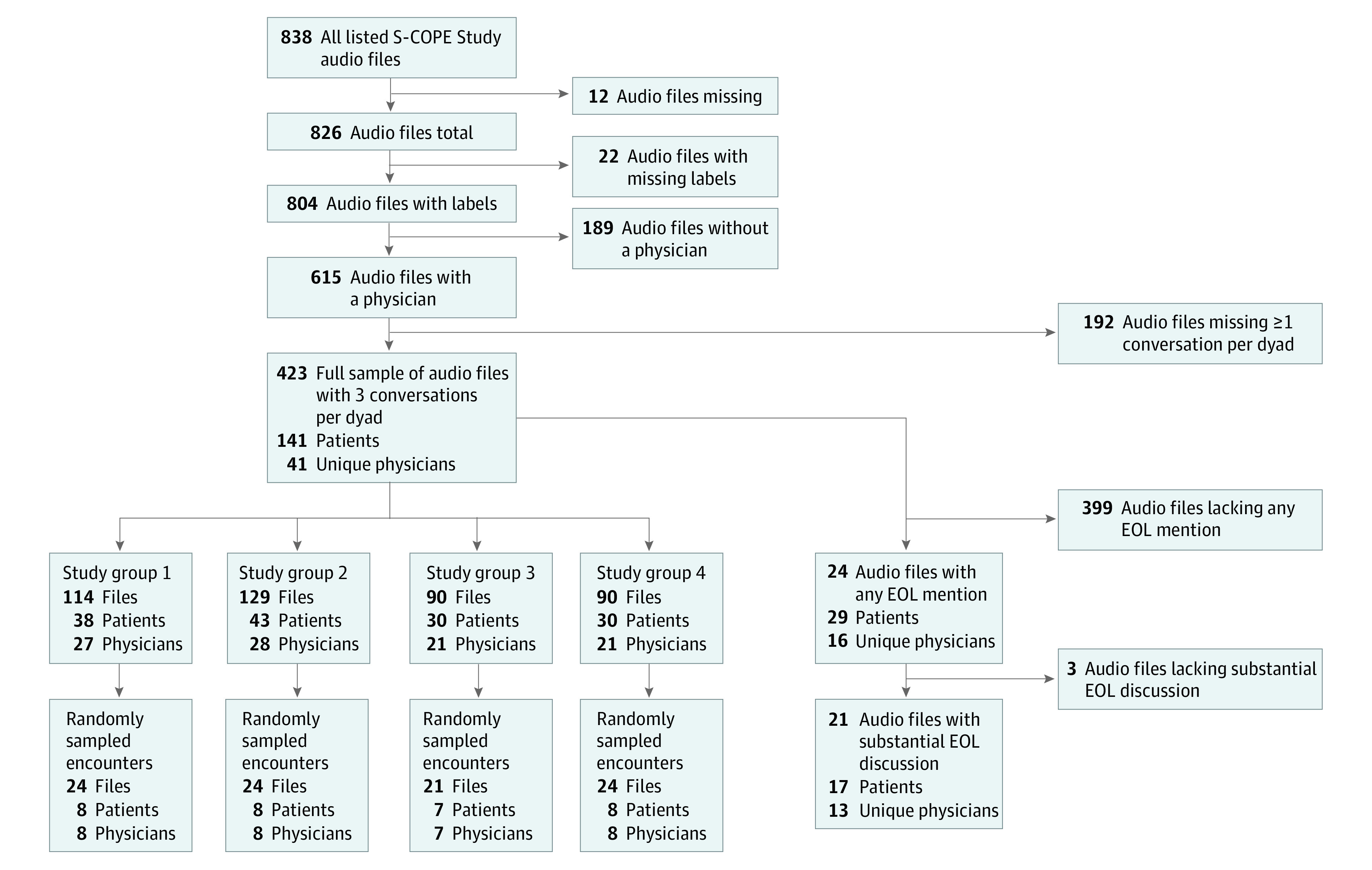
Flow Diagram of Audio-Recorded Encounters: Inclusion and Exclusion Criteria EOL indicates end of life; S-COPE, Studying Communication in Oncologist-Patient Encounters.

We used qualitative content analysis to identify, count, and categorize salient features of patient-oncologist encounters using both deductive and inductive coding.^15^ After content analysis, we performed iterative, thematic analyses of the coded data to identify emergent themes from the encounters. The codebook, which was developed both deductively and inductively, is summarized in Table 1. A multidisciplinary team developed an initial codebook to identify instances of ACP, palliative care, hospice, and discontinuation of disease-directed treatment through iterative review of 10 encounters. Because there were few EOL discussions, the codebook was further developed to identify instances where opportunities were missed, drawing on prior work regarding missed opportunities for ACP in outpatient and inpatient populations.^16,17^ We defined a missed opportunity as lack of practitioner exploration of patient values, goals, or preferences in response to a patient statement regarding cancer progression, death, or disease experience, despite an opening in the conversation where such a discussion would have been appropriate, or even necessary. Missed opportunities were identified and described using the codes from the physician statements, patient of family statements, and physician responses sections of the codebook (Table 1).

**Table 1.** Codebook for Actual and Missed Opportunities to Discuss Advance Care Planning, Palliative Care, Treatment Discontinuation, Hospice Care, and After-Death Wishes

Code	Definition	Example quotation
EOL topics		
Advance care planning	Discussion of advance care planning: identification and documentation of patients’ goals and values for their future medical care	Oncologist: The important thing is if you felt bad at some point and you had to come into an emergency department or something like that—because remember, we have spoken about you didn’t want to have chest compressions and be on a breathing machine and stuff.
		Patient: Right, right.
		Oncologist: And they are going to ask you those things. They have to ask you those things. Just be forthright with them and tell them. So they don’t do anything to harm you against your will.
		Patient: In fact, maybe next time I’ll make sure it’s on—
		Oncologist: Well, it’s one of the things that we do in the hospice program is that they’ll fill that out immediately. But we’re glad to do it as well. We can get you—I’ll be glad to fill that out for you so that we have it.
Palliative care	Discussion of palliative or supportive care services or explicit referral	Oncologist: One way, I think that may help, is we’ve started a new clinic with a group of doctors we call palliative care specialists, that really focus on symptoms, symptom management, pain, depression, whatever it may be to try to see what we can do to help with that part of the equation. Treating the person, not necessarily treating the disease. That’s really what they’re focused on. I have to think I am that way too, but obviously we need people that are really expert, and have expertise in that area.
Hospice care	Discussion of hospice care or explicit referral	Patient: I’m eligible for any of the—what’s the—
		Oncologist: Hospice?
		Patient: Hospice.
		Oncologist (male): I think that’s a—you and I have spoken about that before. We talked about it a few months ago as being perhaps—you’re feeling so good now, that’s a very good option. Because the idea of that is to maintain you feeling as well as they can for as long as they can. What’s nice about it is you don’t have to come in here all the time. Because they are our extension. They will report to us problems you have. We work together to go through them. You get to be home, travel, do what you want as much as you want. I think that’s a very good thing. It’s usually saying, the thing is, that you have less than six months to live. I think if we don’t treat you, you know, you always ask me to tell you.
Discontinuation of disease-directed treatment	Discussion of discontinuation of treatment; includes treatment break or holiday	Oncologist: We’ve given you very good chemotherapy. You have had some side effects from it. By and large, you have continued to live your life with some issues, but I think at this point you would be better off if we take a step back and we say, “Let’s see if this is going to grow. Let’s see if this is going to bother you,” okay?
		Patient: Mm-hmm (affirmative).
		Oncologist: Instead of going after it with more chemotherapy.
After-death wishes	Discussion of postmortem preferences, such as for handling of the body or for services of remembrance	Patient: I always thought that I wanted to be cremated and now I don’t. And my decision is this, and I don’t know how to go about it. I would like to leave my body for research.
Initiation of EOL discussion		
Patient	Initiation of EOL discussion by patient	Patient: I think I know what my decision is going to be. Which is probably going to stay—just going to—
		Oncologist: The hospice and palliative care?
		Patient: Go to hospice full-time and whatever.
Family or caregiver	Initiation of serious illness conversation by family member or caregiver	Family member: Well, could both y’all talking...can I say one thing? That be open with her and you understand, whatever trial you take, what kind of consequences you can reap from trying to...with the normal things that happen to that, as opposed to giving up quality of life.
Oncologist	Initiation of serious illness conversation discussion by oncologist	Oncologist: I know you’ve been treated with eight lines of therapy, you always have to reassess, is additional chemotherapy the right thing to do? And that’s based on your quality of life and how well you’re doing.
Goals and values		
Patient	Statement by patient of values and goals for treatment	Patient: Yeah. Well, right now, I don’t know. I mean, my feeling is, I mean, like you said, I’ve, I’ve been going on chemo since I think July to fourth or somewhere around here, running chemo all this time. And it really hasn’t done me any good. I mean, I can’t say that. I mean I—
		Oncologist: Well you don’t have to hurt my feelings or you know, you’re not hurting my, I understand where you’re coming from.
		Patient: It hasn’t grown any but it hasn’t shrunk where I wanted to get operated on and everything else and I’m to the point right now, I like to take a little rest.
		Oncologist: I have no problem with that. I have no problem with that.
Family or caregiver	Statement by family member or caregiver of patient’s values and goals for treatment	Family member: And we don’t want to get into the situation where we say, “Okay, what can be done for her?” And he is asking, “How do you feel? Oh, you look good. You can take it. You can take more,” and [patient] says, “Yeah, but I want a quality of life too,” and he immediately seems to recoil and give less, you know? I don’t want somebody to say, “It doesn’t matter anyway. Let’s give her as long as she feels good.” I think we want to extend life as long as possible.
Oncologist explores	Oncologist explores patient’s values and goals for treatment	Oncologist: Now, what’s the reasonable thing to do? Let’s take a step back, and ask what our goals of therapy are? What are we trying to achieve?
		Patient: To make me feel better and me live longer.
		Oncologist: You want to feel good, and you want to live longer. Exactly right. I agree with you 100%.
Oncologist states	Oncologist declaratively states next steps for treatment or care trajectory	Oncologist: But to be honest the, the extent that the neurologic deficits, you know the fact that he’s really essentially paralyzed on the left is also you know gonna limit the, limit the treatment options. So I, I think that right now the goal, you know the goal of therapy, you know realistically is going to be to try and keep things the way they are now neurologically for as long as we can and you know I, I don’t think, you know we want to make, we, we, we want to be careful in making decisions about um, the goals of care and when to stop treatment. You know very cautiously in the setting of major depression because some of his, the quality of life issues I think are depression-related, but I think we, we do need to consider and I think you know he needs to talk with you about if the, if the goal of treatment is to keep things the way they are now and it’s unlikely that we’re going to make things better is that an adequate goal that he wants to accept the potential risks of treatment um, you know to prolong survival in his current state? You know I think he is, he is interacting with family, he’s, you know he’s, he knows what’s going on, you know I would, I would usually say in situations like this that you know there’s still sentient, you know conscious being where the person is potentially able to enjoy being with their family and being able to enjoy being alive and I think that we, we usually wouldn’t throw in the towel at this, at this point in terms of further um, further antitumor treatment.
EOL discussion content		
Anticipatory guidance	Proactive education approach that prepares patients for what they should expect in the coming months or years; includes signposting	Oncologist: Here’s my concern—when are you going to Las Vegas?
		Patient: It depends on the—it’s going to be only a three-day trip over the weekend.
		Oncologist: To be honest with you, if I was going to take this trip, I would take it sooner. I think what’s going to happen is sometime in the next few months—I don’t think it’s going to be in the next couple of weeks, but it may be in the next few months what’s going to happen and with these liver enzymes creeping up is going to tell us is that those liver metastasis are going to cause a problem where you no longer feel better. You start feeling worse because of liver failure.
		Patient: What symptoms are that? Probably _____.
		Oncologist: Well yeah, exactly. So what are the symptoms? Feeling tired, fatigued, loss of appetite, and sometimes people get jaundiced. You know, get jaundiced again.
		Patient: That’s what happened the first time, yeah. Right, okay.
		Oncologist: So we’d like to—if we’re going to think of treating you – and this is important [patient’s name]. If we think we’re going to treat you, it’s better to treat you before you get to that point than after that point.
Discussion: trade-offs	Discussion of trade-offs between possible treatment trajectories	Oncologist: If you can’t remove it, then we’re always weighing the risks of benefits of what we’re doing. Okay. You know, if we’re making it better, for sure, we get to continue. If we’re keeping it where it is and you’re and you’re miserable or really beat up, you have to make these hard decisions about is it worthwhile continuing doing this or am I better off saying, listen you know, there are things I want to do and I don’t want to feel like this. I can’t make that decision for you. No one else can make that decision for you. I mean, you could talk to your wife you and you can talk to your family and your children, but to be honest with you, when push comes to shove [patient’s name], you’re the only person who’s really going to be able to make that decision.
Discussion: postpone	Discussion or decision postponed to a later time	Patient: And I might be in better shape because I’m not taking the chemo.
		Oncologist: So that’s why I wanted to know, what are your priorities?
		Patient: Well, let’s go another six weeks without the chemo and see what happens.
		Oncologist: In terms of how you feel.
		Patient: Of how I feel. And let’s see where I am with the—see how the cancer grows. You can—you know, another six weeks. I don’t know if I’m due for another CT scan.
		Oncologist: No, you wouldn’t be in six weeks. But we could follow the tumor marker. We can evaluate how you’re feeling. Absolutely.
		Patient: Right. And then I’ll come back and we’ll talk about it again. Maybe I can go on the chemo if I’m feeling up to it…and so forth.
Physician statements		
Physician concern	Physician expresses concern about the future	Oncologist: I worry about waiting three months with nothing else.
Optimistic future talk	Physician presents the best possible scenario for treatment or prognosis with no realistic expectation setting	Oncologist: And if it’s working, we keep it going. And I’ve had patients on this drug for as long as four and a half years.
Failure to elicit preferences	Physician fails to elicit patient preferences when discussing disease progression	Oncologist: Here are the reports of the chest, and the neck, and the abdomen… Again, the best way of judging this is how people are feeling. I think you’re feeling okay.
Patient or family statements		
Pertaining to the future	Patient or family talks about future care, health, or plans related to EOL	Patient: I was wondering if I could delay for one more month to see if I can get some more energy back.
Worry or concern		
Direct	Patient or family expresses direct worry or concern about future care, health, or plans related to EOL	Family: [Our daughters are] just really so worried about him.
Indirect	Patient or family expresses indirect worry or concern about future care, health, or plans related to EOL	Patient: And you know I really hate to see my family go through everything they’re going through over this, it drives me crazy.
Question		
Direct	Patient or family asks a direct question about disease progression or prognosis	Family: So, you think he’ll live, huh?
Indirect	Patient or family asks an indirect question about disease progression or prognosis	Patient: One, one other things was, I guess, we were looking for, uh, options other than going on this trial.
Physician responses (to patient or family statements)		
Present	Physician has an adequate or complete response	Oncologist: While it may be slowing the disease down, you pay a price every time you get chemotherapy. And the question is, is the price worth it? Because it isn’t curing the disease and it beats you up. So you know, no one can make this decision for you other than you.
Partial	Physician has an incomplete or inadequate response	Patient: And you think the radiation, uh, would probably be necessary?
		Oncologist: Mm-hmm.
Avoidant	Physician changes the subject in response	Oncologist: Well yeah it’s, it’s never easy dealing with tough diseases like this. Well let me check on you.
Absent	Physician has no direct response	Family: Hopefully we have good news today.
		Oncologist: Okay.
Denial	Physician actively negates the patient’s statement	Patient: When somebody tells you about stage 4 cancer, I mean, everybody looks at you like, well, you know, you’re fixing to die. I mean, really, people do.
		Oncologist: A lot of people live for years with stage 4 cancer.

Abbreviation: EOL, end of life.

One investigator (K.E.K.) independently reviewed the entire random sample for EOL discussions and missed opportunities, whereas a second investigator (O.A.S.) independently reviewed 65% of the sample. All instances of missed opportunities and associated codes were iteratively reviewed and jointly agreed upon by both investigators. For the remainder of the full sample, 2 investigators (S.S.A. and K.E.K.) trained 6 coders (J.H.J., L.A.H., O.C.B.B., and 3 others who were not authors of this article) to listen to and flag the 330 audio-recorded encounters for discussion of ACP, palliative care, treatment discontinuation, and hospice care; through this process, we added discussion of after-death wishes as an EOL component (older African American patients often interpret *EOL* as referring to after death^18^). Three investigators (K.E.K., A.E.B., and G.F.M.) adjudicated all flagged encounters. Two investigators (K.E.K. and O.C.B.B.) further developed the original codebook (initially based on a small number of EOL discussions) to describe the character and content of these conversations. A multidisciplinary team (K.E.K., O.C.B.B., and G.F.M.) applied the codebook to all encounters in the full sample with any EOL discussion. Codes applied to this sample of encounters can be found in the EOL topics, initiation of EOL discussion, goals and values, and EOL discussion content sections of the codebook (Table 1).

### Statistical Analysis

Investigators were blinded to study group and demographic characteristics other than sex. Throughout independent review of all transcripts, the team iteratively discussed differences in coding and came to consensus; for this reason, intercoder agreement was not assessed quantitatively. Upon completion of coding, the team iteratively reviewed coded excerpts from encounter transcripts to identify emergent themes. Separate thematic analyses were conducted for encounters that included EOL discussions and encounters that included missed opportunities, as each sample yielded different themes. These are presented sequentially in the Results section. Data were analyzed using Altas.ti statistical software version 8.0 (Scientific Software Development).

## Results

Of 141 unique patients in the full sample, 54 (38.3%) were women and 123 (87.2%) were White. Of 39 unique oncologists, 8 (19.5%) were women, and 34 (82.9%) were White. The mean (SD) age for both patients and oncologists was 56.3 (10.0) years. We summarize sample characteristics in Table 2.

**Table 2.** Demographic Characteristics of the Full Sample of Patients and Oncologists

Characteristic	Individuals, No. (%)
Patients	
No. (%)	141 (100.0)
Sex	
Male	87 (61.7)
Female	54 (38.3)
Race/ethnicity	
White	123 (87.2)
Black or African American	9 (6.4)
Asian or Pacific Islander	2 (1.4)
Hispanic	1 (0.7)
Other^a^	7 (5.0)
Oncologists	
No. (%)	41 (100.0)
Sex	
Male	32 (78.0)
Female	8 (19.5)
Race/ethnicity	
White	34 (82.9)
Black or African American	1 (2.4)
Asian or Pacific Islander	4 (9.8)
Hispanic	2 (4.9)
Other^a^	1 (2.4)
Age, mean (SD)	56.3 (10.0)

^a^ Other includes both individuals who selected American Indian or Alaska Native and those who selected *other* with a free-text option to specify.

In the full sample (141 dyads and 423 encounters), 21 of 423 encounters (5%) included discussions of ACP, palliative care, treatment discontinuation, hospice care, or after-death wishes. Three dyads had more than 1 encounter with an EOL discussion, translating to 17 of 141 dyads (12%) with at least 1 EOL discussion. These 17 dyads included 13 of 39 oncologists (33%). In our subgroup analysis of the random sample (31 dyads and 93 encounters), 5 of 93 encounters (5%) included EOL discussions and 35 of 93 encounters (38%) included missed opportunities. When accounting for dyads with missed opportunities over multiple encounters, 19 of 31 distinct dyads (61%) included missed opportunities. These 19 dyads included 14 of 22 oncologists (64%). All 22 oncologists in the random sample were represented in the full sample. Accounting for overlap, 23 oncologists had either a missed opportunity or actual EOL discussion; only 4 of 23 (17.4%) had both. Our analysis of EOL discussions found 3 emergent themes describing ways in which oncologists successfully navigated opportunities to explore patient goals, preferences, and values related to EOL: (1) reevaluating treatment options in response to patients’ expressions of concern, (2) honoring patients as experts on their goals, or (3) using anticipatory guidance to frame treatment reevaluation.

### Reevaluating Treatment Options in Response to Patients’ Expression of Concern

When capitalizing on opportunities to discuss EOL, oncologists outlined trade-offs between disease-directed treatment continuation and discontinuation. For example, they that acknowledged more chemotherapy could help achieve the goal of prolonging survival by slowing the cancer’s growth, yet also recognized the patient would likely experience discomfort due to treatment adverse effects. These adverse effects proved prohibitive for many patients, preventing them from attending important events, vacationing with family, or “doing all the things they want to do.” At least 1 oncologist refuted the notion that more chemotherapy directly correlates with prolonged survival (in advanced ovarian cancer), citing relevant literature: “There was no differen[ce] in terms of the outcome of how long people lived.” The downsides of excessive chemotherapy (eg, adverse effects and potential futility) were prominent features in treatment discontinuation discussions, emphasizing quality of life over survival. Ultimately, the patients, rather than their oncologists, determined whether they could tolerate further treatment or whether the adverse effects were substantially interfering with their quality of life.

### Honoring Patient as Experts on Their Goals

Oncologists who engaged in successful EOL discussions relied on patients to guide treatment decisions, demonstrated by the way they asked their patient questions. One oncologist initiated one such conversation by asking, “How would you feel? What would you like? What was the goal that you would like to attain?” By opening with the patient’s goals at the forefront, this oncologist allowed the patient to shape the subsequent conversation, using their goals as guiding pillars for any resulting recommendations. Furthermore, the use of a hypothetical gave the patient an opportunity to explore their feelings toward treatment discontinuation in an approachable way that did not demand immediate commitment. The oncologist acted as a facilitator, creating an environment for reflection, whereas the patient led the decision-making. In addition, oncologists often referenced the patient, rather than the cancer, as the subject, indicating the patient’s quality of life as the primary focus. As one oncologist said to his patient, “The scan doesn’t tell the story. You tell the story.” The oncologist’s guidance regarding treatment decision-making recognized the patient’s symptom burden as the driving factor, directly linking the treatment decision to the patient’s quality of life. In this manner, the oncologist emphasized treatment of the person, not the disease.

### Using Anticipatory Guidance to Frame Treatment Reevaluation

When discussing next steps, oncologists often turned to anticipatory guidance to help patients frame their preferences, goals, and values for EOL treatment. Anticipatory guidance was used to indicate a potential, future time when patients would have to make decisions favoring quality of life over disease-directed treatment. At this stage, oncologists offered signposts to indicate to patients may want to stop treatment, meaning they would “have to think in terms of a balance between quality of life and a desire to treat the cancer.” Clear signposts—such as, “if you’re not feeling well and you’re not tolerating things and you’re beginning to lose weight and have more problems with energy, and pain is becoming more of an issue”—allowed patients to imagine (and later recognize) a point in the future when they would need to consider discontinuing treatment. Oncologists demonstrated anticipatory guidance by maintaining transparency with their patients when they deemed treatment to be no longer beneficial, giving their patients an opening to opt out of further treatment. One oncologist noted that continuing treatment could cause more harm than good, stating, “I would be very reluctant to treat you. I think it would be wrong. It’s always better to be able to treat and gives us some hope. But I don’t want to give you false hope.” This oncologist could not maintain an exploratory attitude toward continued treatment in good faith and was honest about this anticipation with the patient, which opened a window to discuss EOL possibilities.

### Missed Opportunities

Our analysis of the random sample identified 3 emergent themes that described ways in which oncologists deflected EOL discussions, leading to missed opportunities to elicit or explore patient goals, values, and preferences: (1) responding inadequately to patient concerns related to disease progression or dying, (2) using optimistic future talk to address patient concerns, or (3) expressing concern over treatment discontinuation.

#### Responding Inadequately to Patient Concerns About Disease Progression

When patients or caregivers expressed concern over disease progression or dying, they were often met with partial, avoidant, or absent responses from their oncologists. When oncologists did respond, they often deflected concerns, rather than using them as openings to explore patients’ goals, values, or preferences for care, according to best practice in patient-practitioner communication.^19^ In one such instance, a practitioner responded to concern over a patient’s prognosis by joking, “You’ll live until you die.” Although humor can help build rapport, and the quip did explicitly name death, the practitioner could have probed the sentiments underlying the family member’s question. Another oncologist missed an opportunity for a discussion on goals of care, giving no response when the patient indicated she was fearful of the prospect of living for years with stage IV cancer. Exploring this statement could have led to a fruitful discussion of disease burden, treatment decision-making, and EOL care preferences.

#### Using Optimistic Future Talk to Address Patient Concerns

Optimistic future talk describes oncologists’ responses to patients’ concerns regarding disease progression or dying. Instead of addressing patients’ concerns directly, oncologists often shared anecdotes of patients who exceeded average life expectancy. When 1 patient expressed worry about the future, she was met with optimism from her oncologist, who said, “A lot of people live for years with stage IV cancer.” The patient’s family member then offered, “He’s got a patient that lives—has lived ten years, right, that one guy?” Here, the oncologist used the family member’s comment to avoid a realistic conversation about prognosis.

#### Expressing Concern Over Discontinuing Treatment

Oncologists frequently expressed concern over treatment discontinuation, often referred to as a holiday or break. In one instance, a patient asked his oncologist if he could delay treatment to regain some energy. His oncologist expressed concern at this idea, saying, “What I would be worried is that, if you continue to not take it and the disease progressed, you might actually get more tired.” Although the oncologist justified his decision by linking the patient’s goal to his recommendation, he missed an opportunity to discuss the patient’s quality of life and treatment goals, at the mention of a symptom so burdensome the patient was interested in treatment discontinuation. Often, oncologists folded statements of concern about discontinuation into discussions of future treatment, leaving no room to discuss discontinuation. In another encounter, a patient requested a treatment switch, to which his oncologist voiced concern over his being without treatment during the washout period. The oncologist then immediately recommended another treatment option that did not require a washout period. By declaratively indicating next steps, the oncologist missed an opportunity to discuss EOL options, which may have been appropriate given the patient’s disease progression.

## Discussion

In this secondary analysis of outpatient oncology visits, EOL discussions were rare and missed opportunities for these discussions were common. When oncologists did discuss EOL, they framed it around trade-offs, anticipatory guidance, and acknowledging patients as experts. In these instances, oncologists adeptly responded to patients’ emotions and expressed empathy. In contrast, missed opportunities for EOL discussions were characterized by failure to acknowledge patients’ concerns over disease progression, dying, or burdensome adverse effects; oncologists often responded to such concerns with optimism about prognosis or pessimism about treatment discontinuation. Although we recognize that not every patient or appointment may necessitate an EOL discussion, all patients in this study had stage IV malignant neoplasm and their oncologists had previously acknowledged that they “would not be surprised if they were admitted to an intensive care unit or died within one year.” Despite the urgent necessity of EOL discussions within this population, we found far more missed opportunities than actual discussions in this analysis.

Considerable prior research demonstrates the infrequency of EOL discussions; only approximately 10% of patients with advanced cancer will ever discuss EOL care with their oncologist.^10^ Decades of work devoted to communication skills training for oncologists, including training in noticing and responding to emotion cues, have not been particularly successful in shifting these figures.^20,21,22,23,24,25,26^ Evidence suggests that physicians may be uncomfortable discussing EOL topics and, thus, avoid them.^27^ In addition, emotional distancing due to physician stress or burnout may decrease the likelihood that physicians pick up subtle cues from patients regarding concerns about their disease.^28^ Because many oncologists normalized patients’ worries, glossed over difficult topics, or avoided direct exploration of EOL preferences, they may have lacked comfort and willingness to engage in difficult conversations about death and dying. It is also possible some oncologists believed they were sparing patients from conversations they were not yet ready to have, or preserving hope—albeit, potentially to the patient’s detriment—of continuing treatment. In this vein, some oncologists may have felt a sense of denial as to their patients’ disease progression, particularly after developing close relationships with their patients.

The purpose of the parent clinical trial was to improve communication between oncologists and patients, thus encouraging goal-concordant care. Oncologists may feel uncomfortable prognosticating life expectancy in a way that opens the door to EOL discussions,^29,30,31^ and prognostication continues to grow increasingly difficult as new treatment options emerge. Although it is comfortable for practitioners to discuss best-case scenarios and hopeful anecdotes, focusing on them creates challenges for future medical decision-making.^32^ Successful EOL conversations included anticipatory guidance about trade-offs of quality of life and symptom burden; these topics may be less uncomfortable for oncologists to broach and may be useful strategies for starting these conversations. Furthermore, oncologists who engaged in these conversations were meeting patients’ emotional concerns with understanding and empathy. We posit that the gap between EOL discussions and missed opportunities may center around affective work regarding empathy and tolerance of emotional discomfort, rather than cognitive work regarding future planning or conveying treatment information.

### Limitations

This study has several limitations. Despite the number of included encounters, we acknowledge that dyads may have discussed EOL preferences before the recorded conversations. As such, the frequency of EOL discussions may be underreported. Furthermore, it would be infeasible to assume that oncologists take advantage of every opportunity to discuss EOL, all of which we identified in retrospect. These data were collected as part of a clinical intervention study designed to improve how patients express emotion to their oncologists. There was no intervention for the oncologists in this trial. Thus, there might be more expressions of patient emotion in these data, but this should not have influenced how oncologists responded. In addition, these data are 7 to 11 years old; it is possible that outpatient oncology communication has changed since then. However, recent data indicate that the frequency of EOL discussions remains low among patients with advanced cancer.^33^

## Conclusions

Early initiation of discussions regarding patient goals of treatment and preferences for EOL care may improve EOL outcomes for patients.^11,34,35,36,37,38^ Given that decades of work have aimed to improve oncologist communication of EOL topics, it is concerning these opportunities for discussion are still largely unheeded. Furthermore, missed opportunities to discuss ACP, palliative care, hospice care, treatment discontinuation, and after-death wishes allow aggressive, burdensome, and expensive EOL treatment to continue unchecked. Future efforts must address barriers, such as oncologists’ fear of upsetting patients and discomfort engaging in EOL discussions, and challenges inherent to providing a time-sensitive prognosis, in tandem with the need to respond to patient emotions and express empathy.
